# Draft Genome Sequence of Enterobacter oligotrophicus, Isolated from the Microbiome of a Lizard in the Caribbean

**DOI:** 10.1128/MRA.00602-21

**Published:** 2021-09-02

**Authors:** Matthieu Pot, Célia Ducat, Yann Reynaud, David Couvin, Séverine Ferdinand, Sébastien Breurec, Antoine Talarmin, Stéphanie Guyomard-Rabenirina

**Affiliations:** a Transmission, Reservoir and Diversity of Pathogens Unit, Pasteur Institute of Guadeloupe, Les Abymes, France; b Faculty of Medicine Hyacinthe Bastaraud, University of the Antilles, Pointe-à-Pitre, France; c INSERM, Center for Clinical Investigation 1424, Pointe-à-Pitre/Les Abymes, France; Loyola University Chicago

## Abstract

Here, we describe the genome sequence of ECC486. This Enterobacter oligotrophicus strain was isolated from a wild specimen of Anolis marmoratus speciosus, a lizard endemic to the territory of Guadeloupe (French West Indies). Its draft genome sequence consists of 40 contigs and contains a total of 4,504,233 bp, with a G+C content of 54.1%.

## ANNOUNCEMENT

Enterobacter cloacae complex (ECC) members are isolated from various environments and recognized to be opportunistic pathogens (1). They harbor a chromosomic *ampC* cephalosporinase gene and are capable of exchanging resistance plasmids (1, 2). The introduction of genome sequencing and computational analysis revealed high genomic diversity in this complex, and its classification has been revised several times (3–6).

The present strain was isolated from a wild Anolis marmoratus speciosus specimen in Guadeloupe in October 2018. The lizard was caught in an urban area (16.228871 N, 61.521655 W) (7). A fresh fecal sample was recovered according to the approved procedure and inoculated overnight at 37°C into chromogenic agar (CCA; CHROMagar, Paris, France), after a pre-enrichment step on buffered peptone water (7). A single strain was isolated and initially identified as *Enterobacter hormaechei* (99.9%) by matrix-assisted laser desorption ionization–time of flight mass spectrometry (MALDI-TOF MS) and the associated software (VITEK MS; bioMérieux, Marcy L’Etoile, France) (8).

A pure isolate was obtained after inoculation into fresh CCA medium and cultivated aerobically overnight at 37°C. The genomic DNA was extracted using the QIAamp DNA minikit (Qiagen, Hilden, Germany). After a quality check, a typing experiment was performed by amplifying a partial gene coding for heat shock protein 60 (hsp60) (5, 7). The amplicon was sequenced at Eurofins (286 bp; Eurofins Genomic SAS, Les Ullis, France). BLASTN submission on GenBank version 2.11.0 (https://blast.ncbi.nlm.nih.gov/Blast.cgi) indicated 100% coverage and a maximum percent nucleotide identity with the Enterobacter oligotrophicus reference strain CCA6 (99.30%; GenBank accession number AP019007.1) (9, 10).

The whole genome of strain ECC486 was sequenced to confirm typing observations (see “UD5” in Fig. S1 in the supplementary material for reference 7). This step was performed using a NextSeq 500 system (Illumina; Nextera XT kit library; 150-bp paired-end configuration). Unless otherwise indicated, default parameters were used for all the following software tools. The sequencing step generated a total of 7,081,784 raw reads, which were trimmed and filtered using AlienTrimmer version 0.4.0 (11). *De novo* assembly and annotation were performed using SPAdes version 3.12.0 (“–careful” option) and the Prokaryotic Genome Annotation Pipeline (PGAP) version 5.2 (12, 13). We obtained a 4,504,233-bp long genome sequence assembled into 40 contigs (G+C content, 54.1%). It has an *N*_50_ value of 257,079 bp and a single-copy BUSCO score of 99.5% completeness, for a 94-fold coverage (BUSCO version 5.0.0; QUAST version 5.0.2) (14, 15).

The average nucleotide identity (ANI) was calculated using OrthoANI software version 0.93.1 against a reference panel (3, 16). In addition, determination of closely related validated reference genomes was performed using the later version of the TYGS platform and digital DNA:DNA hybridization (dDDH) (17). The results indicated that ECC486 is closely related to the *E. oligotrophicus* reference strain with an ANI value of 99.12% and a dDDH value of 93.5% (formula *d_4_*) (3, 17, 18). It underlined the usefulness of the hsp60 approach for an initial and less expensive *E. oligotrophicus* screening among this bacterial complex (5, 7, 9). To date, only two associated complete genome sequences are available. These strains were recovered in 1999 (CCA3; GenBank accession number NZ_BNJN00000000.1) and 2016 (CCA6; AP019007.1) from leaf soil samples in Japan (3, 9, 19).

As this bacterium was isolated from a lizard known to live near households, we determined the probability of its being a human pathogen in a “one health” perspective using PathogenFinder version 1.1 (82.2%) (20). Furthermore, in accordance to its previous antimicrobial susceptibility profile, resistance and plasmid analyses only identified a *bla*_ACT_-like gene (selected percent nucleotide identity threshold, 80%; minimum length, 80% for ResFinder version 4.1 and PlasmidFinder version 2.1) (7, 21, 22). Finally, the CRISPRCasFinder version 1.1.2 and PHASTER (upgrade 6) Web tools allowed us to clearly identify a CRISPR/Cas system (2 array sequences with an evidence level of 4; Cas type I-F) and an intact prophage sequence similar to that of strain HK225 (43.6 kb; GenBank accession number NC_019717) (23, 24).

### Data availability.

The NCBI BioProject accession number PRJNA730279 contains the ECC486 annotated genome sequence (GenBank accession number JAHCLV000000000) and the trimmed and filtered reads (SRA accession number SRR14559665). The initial partial sequence encoding hsp60 can be found under GenBank accession number MZ217779.
